# MIL-100(Al) Gels as an Excellent Platform Loaded with Doxorubicin Hydrochloride for pH-Triggered Drug Release and Anticancer Effect

**DOI:** 10.3390/nano8060446

**Published:** 2018-06-19

**Authors:** Yuge Feng, Chengliang Wang, Fei Ke, Jianye Zang, Junfa Zhu

**Affiliations:** 1 National Synchrotron Radiation Laboratory and Department of Chemical Physics, University of Science and Technology of China, Hefei 230029, China; ygfeng@mail.ustc.edu.cn; 2 Hefei National Laboratory for Physical Sciences at Microscale, CAS Center for Excellence in Biomacromolecules, Collaborative Innovation Center of Chemistry for Life Sciences, and School of Life Sciences, University of Science and Technology of China, Hefei 230026, China; wangcl@ustc.edu.cn (C.W.); zangjy@ustc.edu.cn (J.Z.); 3 Department of Applied Chemistry and State Key Laboratory of Tea Plant Biology and Utilization, Anhui Agricultural University, Hefei 230036, China; kefei@ahau.edu.cn

**Keywords:** metal-organic gels, doxorubicin loading and release, pH-responsiveness, anticancer effect

## Abstract

Slow and controlled release systems for drugs have attracted increasing interest recently. A highly efficient metal-organic gel (MOGs) drug delivery carrier, i.e., MIL-100(Al) gel, has been fabricated by a facile, low cost, and environmentally friendly one-pot process. The unique structure of MIL-100(Al) gels has led to a high loading efficiency (620 mg/g) towards doxorubicin hydrochloride (DOX) as a kind of anticancer drug. DOX-loaded MOGs exhibited high stability under physiological conditions and sustained release capacity of DOX for up to three days (under acidic environments). They further showed sustained drug release behavior and excellent antitumor effects in in vitro experiments on HeLa cells, in contrast with the extremely low biotoxicity of MOGs. Our work provides a promising way for anticancer therapy by utilizing this MOGs-based drug delivery system as an efficient and safe vehicle.

## 1. Introduction

Most anticancer chemotherapeutics are controlled at high doses to make up for their premature deterioration and non-specific absorption, which typically results in the development of dose-limited toxicity [1,2,3,4]. As alternatives, slow and controlled release systems for drugs have recently attracted increasing interest [5,6]. On the one hand, the continuous slow and sustained release of small amounts of drug, instead of several large doses, can weaken patient compliance [7]. On the other hand, delivering the drug by controlled release can reduce the side effects, thus improving therapeutic efficiency [8].

Metal-organic framework (MOFs) is a class of crystalline porous hybrids built from metal ions and organic linkers. Its large surface area, tunable pore size, adjustable composition and structure, and versatile functionality character, make it an ideal carrier for slow and controlled release drug delivery [9,10,11,12,13,14,15]. For instance, Horcajada et al. reported that MIL-100(Fe) nanoparticles could load anticancer drugs (doxorubicin, DOX) up to 9%, and a sustained release in phosphate-buffered saline (PBS) within 14 days was observed [16]. Sun et al. reported Cu-metal organic frameworks (MOFs) with mixed ligands, MOFs-2 (40% 1,3,5-benzene tricarboxylate, 60% isophthalic acid) and MOFs-3 (70% 1,3,5-benzene tricarboxylate, 30% isophthalic acid), and their application as the transport vehicles for the delivery of DOX. The MOFs-2 showed the best performance in transport DOX as the consequence of highest loading capacity (95 mg/g). In weak acid solution (pH 5.8), MOFs-2 released 20% DOX in 80 h [17]. Vasconcelos et al. encapsulated the anticancer drug DOX in nano ZIF-8 with a loading capacity of 49 mg/g, which exhibited a progressive release behavior [18]. However, every previous study has its own shortcomings including complicated synthesis routes, intrinsic biotoxicity, low loading capacity, short release time, and poor stability at a physiological pH of 7.4. The shortcomings limit their potential applications in clinical treatment, which requires high qualities of all the performance-indicators above-mentioned.

Metal-organic gels (MOGs), as the emerging carriers, are constructed by the self-assembly of metal ions and suitable ligands through various noncovalent interactions [19,20]. Compared with MOFs, MOGs possess lower density, higher surface area, larger porosity, and can be synthesized in gentle conditions such as cheap and clean solutions, low temperature and short reaction time [21,22,23,24,25,26,27]. Inspired by these outstanding features, herein, we designed a kind of MOG, i.e., MIL-100(Al) (Al_3_O(OH)(H_2_O)_2_(BTC)_2_·nH_2_O) gels synthesized by a facile, low cost, and environmentally friendly one-pot process as the carrier for anticancer drug doxorubicin (DOX). It is encouraging that MIL-100(Al) gels exhibit high performance in all typical indicators. First, they involve a concise synthetic step, large loading capacity for DOX, and low biotoxicity. Second, DOX-loaded MOGs show a slow and sustainable releasing ability and high anticancer efficiency, thus providing a promising approach for clinical anticancer treatment.

## 2. Materials and Methods

### 2.1. Materials and Methods

1,3,5-Benzenetricarboxylic acid (H_3_BTC) was purchased from Sigma-Aldrich (St. Louis, MO, USA). Aluminum nitrate nonahydrate (Al(NO_3_)_3_·9H_2_O) was obtained from Sinopharm (Shanghai, China) Chemical Reagent Co., Ltd., (Shanghai, China). Doxorubicin (DOX) was purchased from Aladdin Biotech Company (Shanghai, China). Other chemicals obtained from commercial suppliers were of analytical reagent. All chemicals were used without further purification.

The powder X-ray diffraction (PXRD) patterns was collected by using the theta rotating anode X-ray diffractometer with Cu target (40 KV, 200 mA) from 2° to 70°. The Fourier transform infrared spectroscopy (FTIR) spectrum was determined using a Magna-IR 750 spectrometer (Nicolet, Madison, WI, USA) in the range of 500–4000 cm^−1^ with a resolution of 4 cm^−1^. The morphologies of the sample were studied using a SIRION200 Schottky field emission scanning electron microscope (FEI, Hillsboro, OR, USA) and JEM-2100F transmission electron microscope (JEOL, Tokyo, Japan) at 200 kV, respectively. Nitrogen adsorption–desorption isotherms were carried out with a Micromeritics TriStar II 3020 adsorption analyzer (Micromeritics, Atlanta, GAM USA) at 77 K. UV-Vis absorption spectra were carried out with a Shimadzu UV-1800 spectrophotometer (Shimadzu, Kyoto, Japan).

### 2.2. Synthesis of MIL-100(Al) Gels

In a typical synthesis procedure, aluminum nitrate nonahydrate (Al(NO_3_)_3_·9H_2_O, 7.6 mmol) and 1,3,5-Benzentricarboxylic acid (H_3_BTC, 5 mmol) were added to 36 mL ethanol [28]. After stirring for 15 min at room temperature to dissolve the solid, the transparent mixture was transferred to a sealed container and heated to 120 °C for one hour. The wet gels were dried in an oven at 80 °C. Finally, the obtained particles were washed by a Soxhlet extractor using ethanol as medium.

### 2.3. Incorporation of DOX

DOX-anticancer drug (10 mg) was first dissolved in 4 mL deionized water and then the MIL-100(Al) gels (10 mg) were added. The suspension was stirred for 24 h in the dark at room temperature. The obtained materials were then centrifuged, washed with deionized water several times, and dried under vacuum condition for further release tests. The supernatant was collected and measured by a UV-Vis spectrophotometer at a wavelength of 480 nm for the calculation of drug loading content and drug loading efficiency. The drug loading capacity was calculated as follows: drug loading capacity = (weight of DOX in MIL-100(Al) gels/weight of nanoparticles). The drug loading efficiency was calculated by: Drug loading efficiency (wt %) = (weight of DOX in MIL-100(Al) gels/weight of feeding DOX) × 100. The delivery concentration of DOX was derived according to the standard curve which was obtained from measuring the UV-Vis adsorption spectra of DOX with different known concentrations in PBS buffer solution (shown in Figure S1) and then by plotting the absorbance as a function of DOX concentration (shown in Figure S2).

### 2.4. Drug Release

The drug release experiment was performed by soaking the sample in PBS buffer solutions (pH = 7.4 and pH = 5.5) at 37 °C. Ten mg of DOX-loaded MIL-100(Al) gels (DOX-loaded MOGs) were suspended into 10 mL PBS solution. The mixture solution was stirred at the temperature of 37 °C in a water bath. At predetermined time intervals, 3 mL of PBS solution was removed and assayed. The volume of each withdrawn sample was replaced by the same volume of fresh PBS solution. The amount of released DOX was calculated according to the absorption analyzed by the UV-Vis spectrophotometer at 480 nm and standard absorbance vis DOX concentration curve. The calibration experiment was performed using different known concentrations of DOX in PBS buffer solution (shown in Figure S1). The derived standard absorbance vis DOX concentration curve is shown in Figure S2.

### 2.5. Cell Cytotoxicity of DOX-Loaded MOGs

HeLa cells were used for cell viability assay. A 96-well plate was used for cell seeding with a total number of about 2 × 10^3^ per well. The cells were first incubated overnight, and then the MIL-100(Al) gels and DOX-loaded MOGs were added in every well with a final concentration ranging from 0.1 μg/mL to 100 μg/mL (0.1 μg/mL, 0.5 μg/mL, 1 μg/mL, 2.5 μg/mL, 5 μg/mL, 10 μg/mL, 25 μg/mL, 50 μg/mL, and 100 μg/mL). Autoclave water was added and treated as the negative control. The cells were incubated with MOGs or DOX-loaded MOGs for 12, 24, 36, 48, and 72 h, respectively. Later, all the medium in the wells were drawn and discharged, and additional MTT solution dissolved in the medium was used to treat the cells for another 4 h. Finally, dimethyl sulfoxide (DMSO) was loaded to replace the medium and dissolve the crystals for further absorbance detection. The absorbance of each well was obtained at the wavelength of 590 nm. Compared to the negative control, the cell viability was calculated. Each sample was repeated five times and the results presented as average values with error bars representing the standard deviation.

### 2.6. Flow Cytometry

HeLa cells (2 × 10^5^) were seeded on a six-well plate and incubated overnight. The next day, cells were incubated with MIL-100(Al) gels (12.5 μg/mL), DOX-loaded MOGs (12.5 μg/mL), and autoclave water overnight, respectively. The cells were washed twice with 1X PBS followed by treatment with 1X trypsin for 5 min before quenching the cells with culture medium. Thereafter, the cells were washed twice with 1X PBS by centrifugation (1000 rpm, 5 min), and 1X ANNEXIN binding buffer (100 μL) was added to the cell together with PI-PE and ANNEXIN V-FITC conjugate. The cells were incubated in the dark for 20 min. Then, they were immediately analyzed with a flow cytometer.

### 2.7. Fluorescence Microscopy Images

The fluorescence microscopy studies were performed on HeLa cells in a confocal dish with a total number of 4 × 10^5^ per dish. MIL-100(Al) gels (200 μL) and DOX-loaded MOGs (200 μL) were added into each dish respectively, to give a final concentration of 12.5 μg/mL and the cells were incubated for 12 h. Thereafter, the medium was removed and the cells were washed three times with 1X PBS. The treated cells were re-suspended in 1X PBS. Then, the ANNEXIN V–FITC conjugate was added (25 μL), and the cells incubated for 15 min in the dark. Thereafter, the ANNEXIN containing PBS was removed and the cells were washed three times with 1X PBS before fixing them with paraformaldehyde solution (4% in 1X PBS, 1 mL). After 20 min, the formaldehyde solution was removed and the cells washed twice with 1X PBS. In the end, the cells were incubated with Hoechst solution (5 μg/ mL, 1 mL) in 1X PBS in the dark for 15 min, and washed with twice with 1X PBS to image.

## 3. Results and Discussion

### 3.1. Morphology and Structure Characterization of MIL-100(Al) Gels

**Figure 1:**
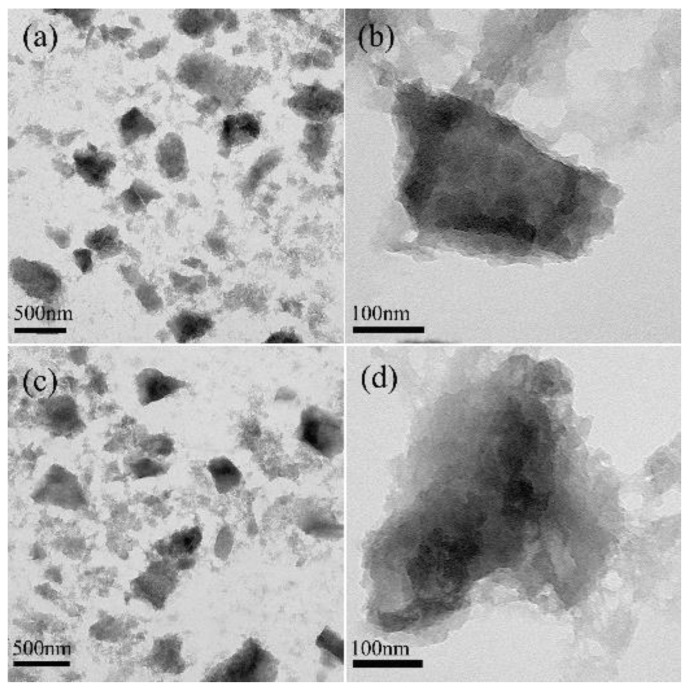
TEM images of (**a**,**b**) MIL-100(Al) gels. (**c**,**d**) DOX-loaded MIL-100(Al) gels.

Transmission electron microscope (TEM) images (Figure 1a,b) showed the irregular structure of the as-synthesized MIL-100(Al) gels. Powder x-ray diffraction (XRD) was applied to identify their microstructure. The result was similar with that of a previous report [29]. As depicted in Figure S3, the pattern for the gel sample revealed a low crystallinity as several broad peaks were observed. However, it also showed that the gel was closely related to the MIL-100(Al) crystal [30]. In each position where a peak appeared for the MIL-100(Al) crystal, there appeared a corresponding broad peak for the gel sample, implying that the gel and the MOF crystal had similar structures. The nitrogen adsorption-desorption isotherm, which was used to evaluate the porous properties of MIL-100(Al) gels, was between those of type-I and type-IV, suggesting the coexistence of micropores and mesopores in the MIL-100(Al) gels sample (Figure S4). The Brunauer–Emmett–Teller (BET) surface area and pore volume of the MIL-100(Al) gels were calculated to be 920 m^2^/g and 0.535 cm^3^/g, respectively. They were slightly lower than those of MIL-100(Al) (1214 m^2^/g and 0.77 cm^3^/g). The similar large surface area and high porosity of MIL-100(Al) gels may arise from the intrinsic nature of the MIL-100(Al) [31]. Thus, the large surface area and high porosity make this material a possible candidate for highly efficient drug loading.

### 3.2. Drug Loading and Release Behaviors

**Figure 2:**
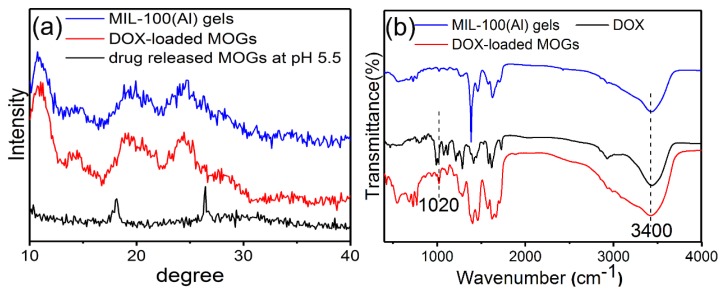
(**a**) Powder XRD patterns of MIL-100(Al) gels, DOX-loaded MOGs, and drug-released MOGs at pH 5.5; (**b**) FTIR spectra of MIL-100(Al) gels, DOX, and DOX-loaded MOGs.

The TEM images of DOX-loaded MOGs (Figure 1c,d) exhibited almost no change in morphology when compared with MIL-100(Al) gels. Figure 2a is the XRD patterns of MIL-100(Al) gels and DOX-loaded MIL-100(Al) gels (DOX-loaded MOGs). Both of them showed similar features before and after the drug adsorption, indicating that the porous structure of MIL-100(Al) gels was retained after the loading of DOX. Figure 2b shows the FTIR spectra of MIL-100(Al) gels, DOX, and DOX-loaded MOGs. The peak at 3400 cm^−1^ was attributed to the O–H stretching of MIL-100(Al) gels. In the FTIR spectrum of DOX, peaks at 1020 cm^−1^ and 3400 cm^−1^ were caused by –NH_2_ torsional vibration and O–H stretching vibrations of DOX, respectively. In the case of the DOX-loaded MOGs, peaks of O–H stretching vibrations overlap were broadened and a new adsorption band at 1020 cm^−1^ owing to the torsional vibration of –NH_2_ from DOX was generated. This FTIR result indicated that MIL-100(Al) gels conjugated with DOX molecules successfully.

It turns out that the loading capacity was reached up to 620 mg of DOX per gram of the sample. This large DOX loading capacity and high loading efficiency (62%) may be attributed to the ultrahigh porosity and enormous internal surface of MIL-100(Al) gels. The driving force of loading of DOX in MOGs may arise from the porous absorption and intermolecular forces. The latter most likely originated from the hydrogen bonds formed between the –NH_2_, –OH groups in DOX and the surface active –OH, –COOH groups in MOGs [6,32].

**Figure 3:**
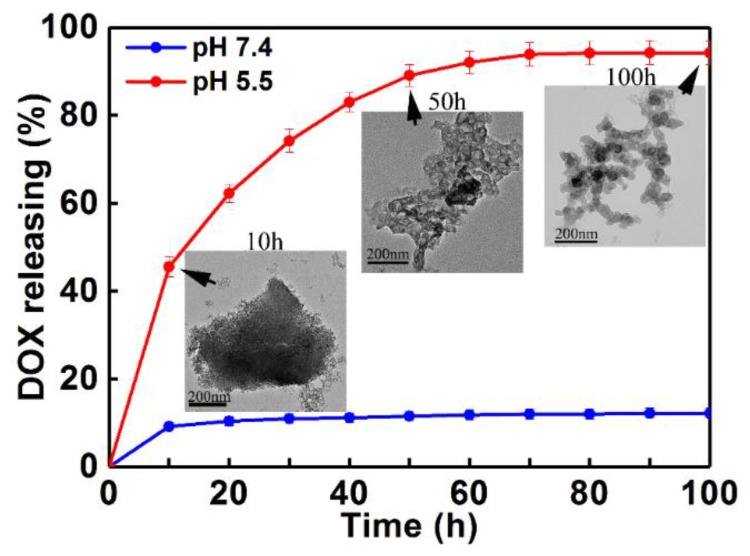
Drug release profiles for DOX-loaded MOGs in PBS buffer solution at pH = 5.5 and pH = 7.4 within 100 h. (Inset are TEM images of DOX-loaded MOGs after 10 h, 50 h, and 100 h in release process at pH 5.5.) Bars denote the standard deviation (±SD, *n* = 5).

The controlled drug release kinetics of DOX from DOX-loaded MOGs were investigated using UV-Vis adsorption spectra in PBS buffer solutions at 37 °C. Figure 3 is the DOX release profiles at two different pH values (pH = 7.4 and 5.5). It can be seen that the release of DOX from DOX-loaded MOGs in pH = 7.4 only reached 10% within 100 h. In contrast, the DOX release rate was significantly increased in pH = 5.5 and this release reached nearly 100% within 100 h. This revealed that under acidic conditions, the drug can be released more easily. Under the weak acidic condition (pH = 5.5), the drug delivery rate gradually decreased with time. Basically, the rate can be clearly divided into three regions: (i) an early rapid release within the first 10 h; (ii) a slow release region in the time range between 10 and 60 h; and (iii) a saturation region after 70 h [33]. The first rapid release was induced by the simple diffusion and dissolution of DOX molecules adsorbed onto the surface of MOGs. The second region revealed a gentle and steady release over a long time due to the desorption, diffusion, and dissolution processes of DOX molecules from channels in the gels to the solution. The last saturated drug release process could be attributed to host-guest interactions between the DOX molecules and the gels. The results revealed that the obtained MIL-100(Al) gels exhibited a high drug loading and long sustained release time under an acidic environment. To further understand the DOX release process from the DOX-loaded MOGs, TEM images were taken from DOX-loaded MOGs after 10 h, 50 h, and 100 h in the release process at pH 5.5 (inset in Figure 3). They revealed the gradual collapse of the MIL-100(Al) gel structure during the procedure. The result was consistent with the XRD pattern of drug-released MOGs at pH 5.5 (Figure 2a), which showed the dissolution of MIL-100(Al) gels in an acidic environment. The reason is that MIL-100(Al) gels self-assemble through multiple non-covalent bonds under preparation conditions such as H-bonds, π–π stacking, electrostatic interactions, and other supramolecular weak interactions [20]. These non-covalent bonds are easily destroyed under acidic conditions, leading to the dissolution of MOGs. The excellent pH-responsive release property may be attributed to the collapse of the MIL-100(Al) gel structure and the reduction of the interaction between DOX and MOGs in acidic conditions (pH 5.5) [34]. In addition, DOX molecules tend to be more hydrophilic at lower pH values [35]. The other possible reason is that the protons can easily penetrate the pores in acidic buffer solution to protonate the amino group of DOX, resulting in the acceleration of drug release [36].

### 3.3. Cell Cytotoxicity of DOX-Loaded MOGs

**Figure 4:**
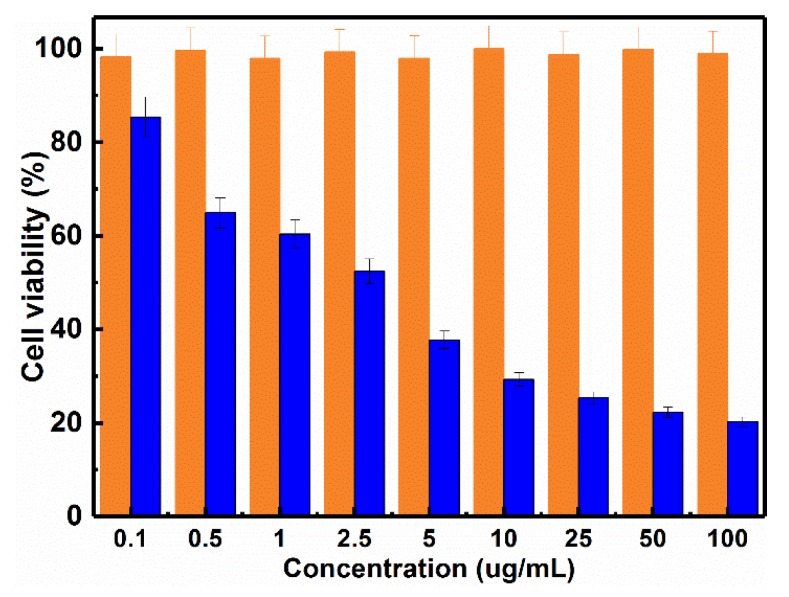
The effect of MIL-100(Al) gels and DOX-loaded MOGs with various concentrations on the cell viability of HeLa cells in 24 h (the orange and blue bars represent the viability of HeLa cancer cells incubated with MIL-100(Al) gels and DOX-loaded MOGs, respectively).

After evaluating the drug loading and release ability of MOGs, in vitro cell viabilities of DOX-loaded MOGs and pure MIL-100(Al) gels on HeLa cells were investigated using the MTT (3-(4,5-Dimethylthiazol-2-yl)-2,5-diphenyltetrazolium bromide) assay. To study the biotoxicity of pure MIL-100(Al) gels and the therapeutic efficiency of DOX-loaded MOGs, HeLa cells were cultured with the DOX-loaded MOGs and pure MIL-100(Al) gels at concentrations ranging from 0.1 μg/mL to 100 μg/mL (0.1, 0.5, 1, 2.5, 5, 10, 25, 50 ,and 100 μg/mL) for 24 h. The results are exhibited in Figure 4. As can be seen, after 24 h incubation with HeLa cells, the pure MIL-100(Al) gels showed no obvious toxicity towards the HeLa cells even at the concentration of MIL-100(Al) gels as high as 100 μg/mL. In contrast, the DOX-loaded MOGs showed high cytotoxicity on HeLa cells. As the concentration of DOX-loaded MOGs increased, the cell viability rapidly decreased. When the concentration of DOX-loaded MOGs reached 25 μg/mL, only ~20% of HeLa cells survived. Therefore, the DOX could be efficiently released from the DOX-loaded MOGs to kill most of the tumor cells, demonstrating that the as-synthesized MIL-100(Al) gels hold great promise for application in the field of drug delivery system for cancer treatment.

**Figure 5:**
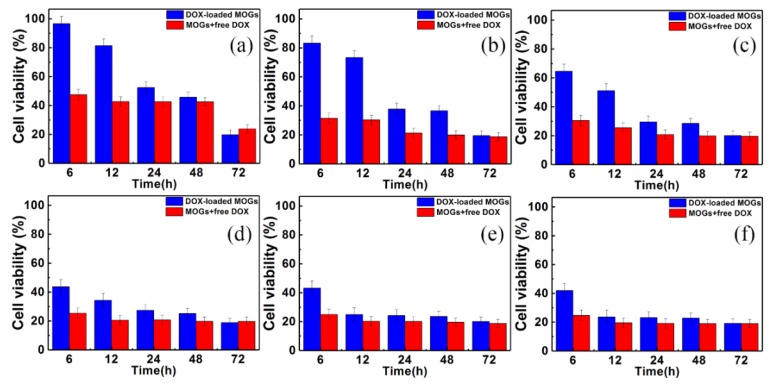
Cell viability of HeLa cells incubated with DOX-loaded MOGs and MOGs + free DOX for different time periods at concentrations of (**a**) 2.5 μg/mL, (**b**) 5 μg/mL, (**c**) 10 μg/mL, (**d**) 25 μg/mL, (**e**) 50 μg/mL, and (**f**) 100 μg/mL.

The in vitro drug release behavior of DOX-loaded MOGs on HeLa cells was also investigated. DOX-loaded MOGs and MOGs + free DOX with different concertation were studied. The results are shown in Figure 5a–f. Accordingly, the viability of cells incubated with DOX-loaded MOGs gradually decreased in the time range of 72 h. This was in contrast with the sudden reduction behavior of the viability of cells incubated with MOGs + free DOX, in all the control experiment groups.

### 3.4. Flow Cytometry

**Figure 6:**
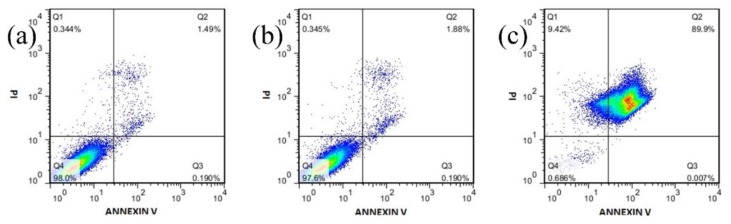
Flow cytometry experiments of HeLa cells when incubated with (**a**) Pure autoclave water as control, (**b**) MIL-100(Al) gels, and (**c**) DOX-loaded MOGs, respectively.

In order to further investigate the apoptosis of the cells, we performed the flow cytometry analysis on HeLa cells with 12.5 μg/mL MIL-100(Al) gels and DOX-loaded MOGs. As shown in Figure 6, almost no necrotic and late apoptotic cells were observed in the control experiment (only containing pure autoclave water) (1.49%) and MIL-100(Al) gels (1.88%), revealing the low toxicity of this MOGs-based material. However, when the DOX-loaded MOGs were added, the percentage of apoptotic cells immediately became prominent (89.9%). These results were in line with the MTT assay and further confirmed that apoptotic cell death arose from the DOX released from DOX-loaded MOGs.

### 3.5. Fluorescence Microscopy Images

**Figure 7:**
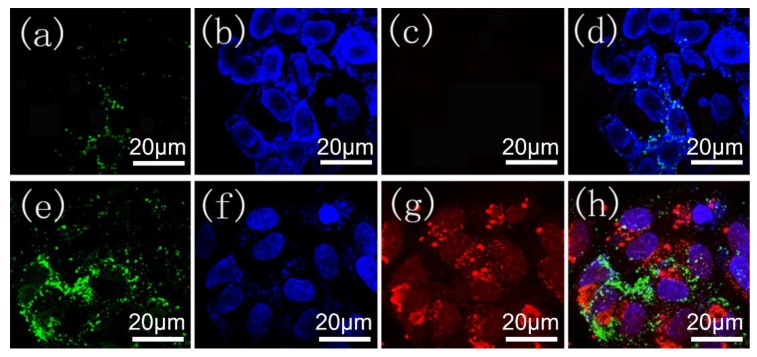
Confocal microscopy images of HeLa cells incubated with 12.5 μg/mL (**a**–**d**) MIL-100(Al) gels and (**e**–**h**) DOX-loaded MOGs, respectively. Blue fluorescence represents the living cell imaging. Red fluorescence represents the released DOX from DOX-loaded MOGs within the cancer cells. Green fluorescence represents the apoptosis of cells. (**d**,**h**) are the merged images of (**a**–**c**) and (**e**–**g**), respectively.

To further confirm the therapeutic efficiency of DOX-loaded MOGs, we performed confocal fluorescence microscopy for HeLa cells incubated with 12.5 μg/mL pure MIL-100(Al) gels and DOX-loaded MOGs for 24 h, followed by staining the nucleus with DAPI and the apoptotic cells with Annexin V-FITC. The results are revealed in Figure 7. Herein, the green fluorescence was attributed to the apoptotic HeLa cells, while the blue and red fluorescence represented the living cell imaging and DOX released, respectively. For the HeLa cells incubated with pure MIL-100(Al) gels, only very small amounts of apoptotic HeLa cells were present (Figure 7c,d). In contrast, for the HeLa cells incubated with DOX-loaded MOGs, a large number of the HeLa cells were apoptotic (Figure 7g,h). This result again demonstrates the high efficiency of the DOX-loaded MOGs in cancer therapeutic treatment.

## 4. Conclusions

On the basis of the methods reported in the previous work [5,6,19,20], we reported on a metal-organic gel (MOG)-based drug delivery system for anticancer therapy, i.e., MIL-100(Al) gels, which were synthesized by a facile, low cost, and environmentally friendly one-pot method. The anticancer drug doxorubicin hydrochloride (DOX) was successfully encapsulated in the MIL-100(Al) gels with high loadings (620 mg/g). Through the control experiments, the fabricated DOX-loaded MOGs were comparable with some previous pH-responsive drug delivery systems [16,17,18]. Specifically, the drug was not released at physiological condition (PBS, pH 7.4), but was released in a controlled manner at acidic conditions (pH 5.5) with approximately 100%, after being delivered over three days. We also conducted in vitro experiments of DOX-loaded MIL-100(Al) gels (DOX-loaded MOGs) toward HeLa cells where it turned out that the DOX-loaded MOGs had excellent efficiency in killing the HeLa cells. The synthetic MIL-100(Al) gels here featured a concise synthetic step, large loading capacity for DOX, and low biotoxicity. Furthermore, the DOX-loaded MOGs showed slow and sustainable releasing ability and high anticancer efficiency. MIL-100(Al) gels exhibited high qualities of all the performance-indicators as above-mentioned, making DOX-loaded MOGs a promising anticancer approach for clinical application.

## Supplementary Materials

The following are available online at http://www.mdpi.com/2079-4991/8/6/446/s1. Figure S1: UV-vis absorption spectra of DOX at different concentrations in PBS buffer solution, Figure S2: Calibration plot of standard DOX concentration, Figure S3: Powder XRD patterns of the as-synthesized MIL-100(Al) gels and the XRD pattern simulated from crystal structure data of MIL-100(Al), Figure S4: Nitrogen adsorption-desorption isotherm of MIL-100(Al) gel measured at 77 K.
