# Supplementary material for: MIL-100(Al) Gels as an Excellent Platform Loaded with Doxorubicin Hydrochloride for pH-Triggered Drug Release and Anticancer Effect
Source: Nanomaterials (Basel). 2018 Jun 19;8(6):446. doi: 10.3390/nano8060446 (PMC6027486; doi:10.3390/nano8060446)
Supplement: Supplementary file 1 [file nanomaterials-08-00446-s001.pdf]

# MIL-100(Al) gels as an excellent platform loaded with doxorubicin hydrochloride for pH-triggered drug release and anticancer effect

**Yuge Feng [1], Chengliang Wang [2], Fei Ke [3], Jianye Zang [2] and Junfa Zhu [1,*]**

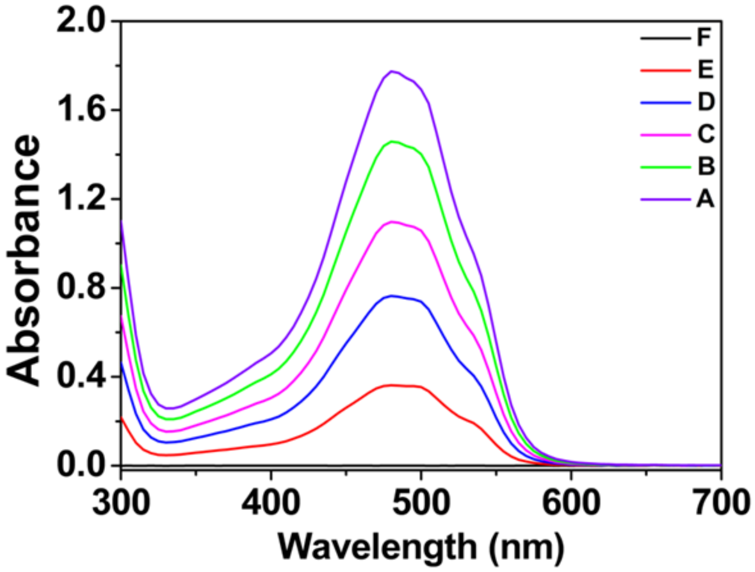


**Fig. S1**. UV-vis absorption spectra of DOX at different concentrations in PBS buffer solution: A (100 μg/mL), B (80 μg/mL), C (60 μg/mL), D (40 μg/mL), E (20 μg/mL), F (0 μg/mL).

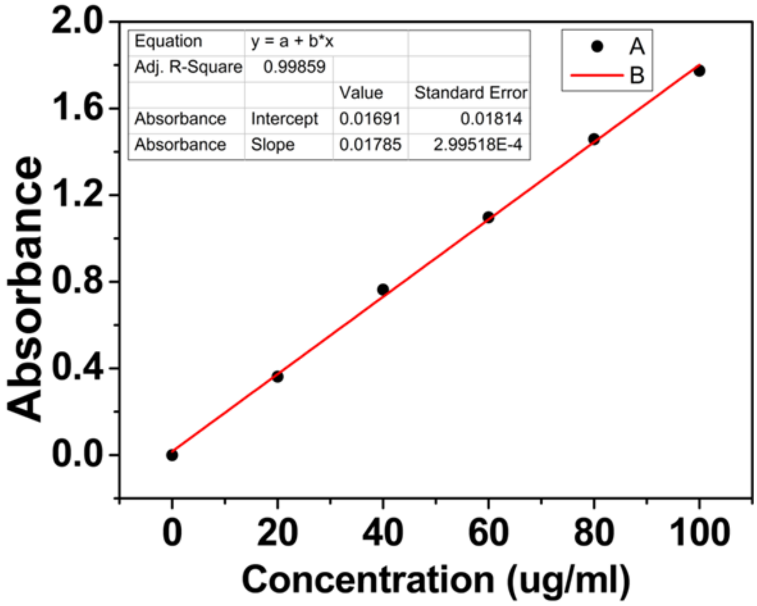


**Fig. S2.** Calibration plot of standard DOX concentration derived from UV-vis absorption spectra in Figure S1.

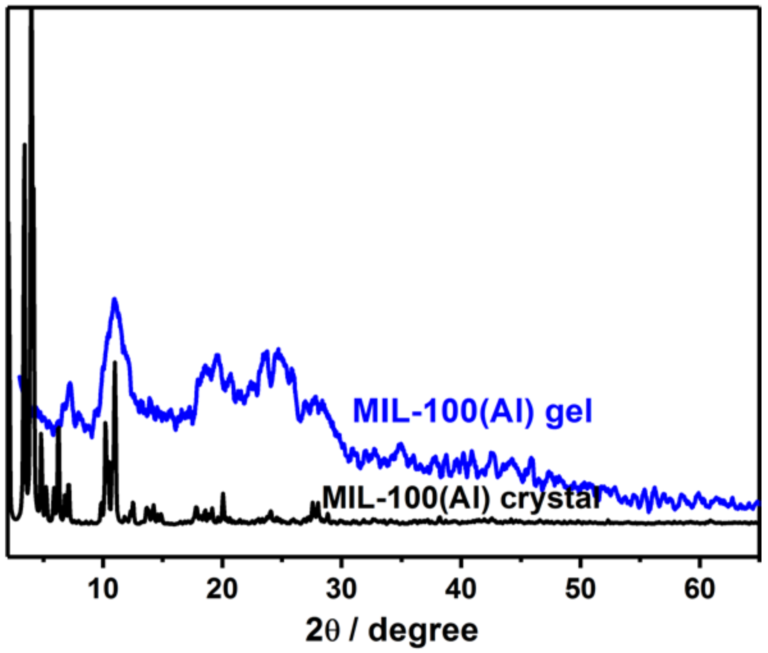


**Fig. S3.** Powder XRD patterns of the as-synthesized MIL-100(Al) gels and the XRD pattern simulated from crystal structure data of MIL-100(Al). The result of the as-synthesized MIL-100(Al) gels agrees well with the simulated result of single-crystal MIL-100(Al).

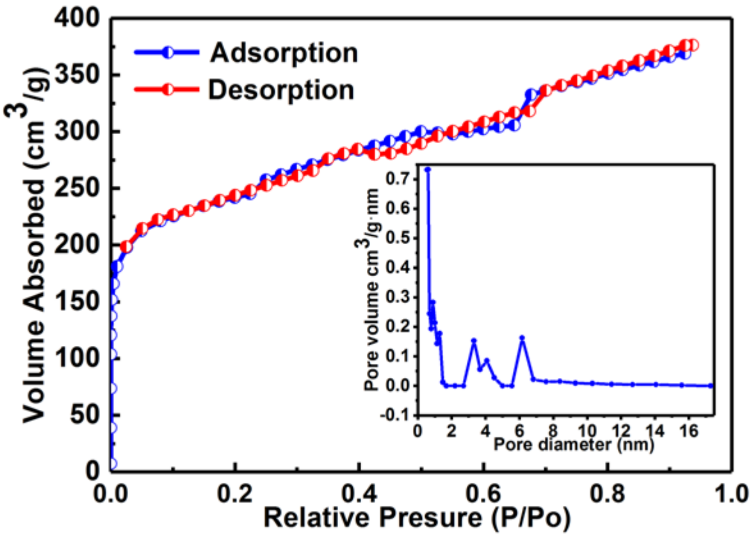


**Fig. S4.** Nitrogen adsorption-desorption isotherm of MIL-100(Al) gel measured at 77 K. The inset shows the corresponding pore size distribution analysis obtained using Discrete Fourier Transform (DFT) method. The BET surface area and pore volume of the MOGs were calculated to be 920 $m^2/g$ and 0.535 $cm^3/g$, respectively. The large surface area and high porosity make this material to be possibly used for highly efficient drug loading.
